# Gain–loss situation modulates neural responses to self–other decision making under risk

**DOI:** 10.1038/s41598-018-37236-9

**Published:** 2019-01-24

**Authors:** Xiangyi Zhang, Shijia Li, Yongfang Liu, Xiyou Chen, Xuesong Shang, Fangzhu Qi, Xiaoyan Wang, Xiuyan Guo, Jie Chen

**Affiliations:** 1 School of Psychology and Cognitive Science, East China Normal University, Shanghai, 200062 China; 2 Key Laboratory of Brain Functional Genomics, Ministry of Education, Shanghai Key Laboratory of Brain Functional Genomics, East China Normal University, Shanghai, 200062 China; 3 Changsha Experimental Middle School, Changsha, 410001 Hunan China; 4 Shanghai Key Laboratory of Magnetic Resonance, East China Normal University, Shanghai, 200062 China; 5 Cognition and Human Behavior Key Laboratory of Hunan Province and Department of Psychology, Hunan Normal University, Changsha, 410081 Hunan China

## Abstract

Although self–other behavioral differences in decision making under risk have been observed in some contexts, little is known about the neural mechanisms underlying such differences. Using functional magnetic resonance imaging (fMRI) and the cups task, in which participants choose between risky and sure options for themselves and others in gain and loss situations, we found that people were more risk-taking when making decisions for themselves than for others in loss situations but were equally risk-averse in gain situations. Significantly stronger activations were observed in the dorsomedial prefrontal cortex (dmPFC) and anterior insula (AI) when making decisions for the self than for others in loss situations but not in gain situations. Furthermore, the activation in the dmPFC was stronger when people made sure choices for others than for themselves in gain situations but not when they made risky choices, and was both stronger when people made sure and risky choices for themselves than for others in loss situations. These findings suggest that gain–loss situation modulates self–other differences in decision making under risk, and people are highly likely to differentiate the self from others when making decisions in loss situations.

## Introduction

Navigating the social environment successfully depends on implementing several operations, including the ability to make optimal decisions under risk for both oneself and others^1,2^. Individuals make decisions for others as often as they make decisions for themselves in daily life^3^. Nevertheless, the process of making decisions for others is not fully understood^4^. Researchers have begun to investigate how people make decisions for others, but inconsistent findings are often observed possibly because of different risky decision-making tasks or situations. For example, Beisswanger *et al*. differentiated between low- and high life-impact decisions, depending on the gravity of the decision’s consequences. They showed that people made more risk-seeking decisions for a same-sex friend than themselves in low life-impact situations (e.g., asking a cute girl to dance at a frat party); however, no self–other difference was found for high life-impact scenarios (e.g., cohabitating with someone)^5^. In a similar vein, Stone *et al*. found that people made more risk-averse decisions for a same-sex friend than for themselves in physical safety contexts (e.g., taking caffeine pills), but made more risk-taking decisions in scenarios involving romantic relationships (e.g., going with a cute guy to a concert)^6^.

Although self–other discrepancies in risky decision making have been observed behaviorally in some domains, involving finance^7^, medicine^8^, and relationships^5,6^, the neural mechanisms underlying such differences remain poorly understood.

Previous neuroimaging studies have mainly focused on decisions for the self. A brain network implicated in risky decision making for oneself has been identified, and includes the dorsomedial prefrontal cortex (dmPFC), anterior insula (AI), thalamus, dorsolateral prefrontal cortex (dlPFC), and parietal cortex^9^. The dmPFC has been implicated in evaluating stimulus risk on a cognitive level^10–12^. For instance, Xue *et al*. showed that dmPFC activation was stronger when participants chose risky as opposed to safe options^11^. Another study showed that such dmPFC activation likely represents the subjective value of monetary outcomes, as well as probability information regarding economic risk^12^. Furthermore, dmPFC plays a role in evaluation of aversive outcomes^13^. Additionally, the AI is active when making decisions for oneself under risk^14–16^. AI activation is stronger when participants choose risky versus sure alternatives^14^. AI activity is correlated with risk prediction and risk prediction errors^16,17^, indicating that this region plays a critical role in facilitating decision biases associated with negative affective influences, such as financial loss or fear. Additionally, dlPFC may be involved in reigning in continued risk-taking, such as modulating risk attitudes^18,19^.

In contrast to the large body of research on neural substrates underlying risky decisions for the self, only one study to date has investigated the neural basis of self–other discrepancies in risky decision making. Jung *et al*.^3^ found that reward-related regions, including the ventral striatum, ventral tegmental area, anterior cingulate cortex, and insula were more active when making decisions for oneself compared with others, whereas the brain regions related to theory of mind, including the temporoparietal junction (TPJ) and posterior cingulate cortex, showed greater activation when decisions were made for others than for oneself. These researchers therefore argue that decisions for oneself may involve mainly the recruitment of affective processes, whereas decisions for others may recruit predominantly cognitive processes.

However, the task used in the study of Jung *et al*. involved mixed gambles that offered a variable chance of either gaining one amount of money or losing the same amount. Specifically, the participants were asked to choose between a low-risk option (i.e., win or lose 10 points) and a high-risk option (i.e., win or lose 90 points). Thus, they win or lose 10 points when a low-risk option was chosen, and win or lose 90 points when a high-risk option was chosen. Obviously, this task cannot separate risky decision-making for gains and losses. More importantly, risky decision making for gains and losses may involve different psychological and neural processes^15,20,21^. Kahneman and Tversky^22^ demonstrated that individuals showed different risk preferences in gain and loss frames. Specifically, people are prone to risk aversion in gain situations but risk taking in loss situations. Using a novel task (i.e., the cups task) that can separate risky decision-making for gains and losses^21,23^, the present study aimed to investigate the neural mechanisms underlying differences between decision making for oneself and for others, in both gain and loss situations.

According to Kahneman and Tversky’s prospect theory, the value function is steeper for losses than for gains^22^, or in other words, the aggravation that one experiences in losing a sum of money seems to be greater than the pleasure associated with gaining the same amount, which suggested that making decisions in loss situations causes more emotional involvement than making decisions in gain situations. Moreover, the degree of emotional involvement in a task may be a decisive factor regarding self–other decision-making differences^5,24,25^. The more one is emotionally involved when making decisions for oneself, the larger the differences in self–other decision making^5,26^. Thus, we expected that the self–other decision-making discrepancies would be larger in loss situations than in gain situations. At the neural level, we hypothesized that the neural response in brain regions implicated in risk processing (e.g., dmPFC, AI) would be stronger when decisions were made for oneself than for others in loss situations as compared with gain situations.

## Methods

### Participants

A total of 28 undergraduate students took part in this fMRI study. All participants were right-handed, had normal or corrected-to-normal vision, no past neurological or psychiatric history, and were not taking any medications. One participant was excluded from the subsequent behavioral and neuroimaging analyses because translational movement parameters exceeded 1 voxel size, and two other participants were also excluded because they indicated doubt regarding the truth of decisions for others on a self-report questionnaire. The remaining 25 participants (14 females, ages 18–25 years, *M* = 20.64, *SD* = 2.06) were included in data analyses. This study was approved by the Ethics Committee of East China Normal University. The methods were carried out in accordance with the relevant guidelines and regulations. The written informed consent was obtained from all participants before scanning.

### Task and procedure

Participants performed a modified cups task^23^, which included gain and loss contexts. In the gain domain, participants were asked to choose between one option that provided a sure gain of ¥10 and another option that offered a designated probability (0.50, 0.33, 0.25, or 0.20) of gaining more (¥20, ¥30, ¥40, or ¥50) or gaining nothing. In the loss domain, they were required to choose between one option that offered a sure loss of ¥10 and another option that offered a designated probability (0.50, 0.33, 0.25, or 0.20) of losing more (¥20, ¥30, ¥40, or ¥50) or losing nothing. In this study, we only adopted gain and loss trials for the following certain combinations in which the levels of outcome magnitude and probability created equal expected values for the sure and risky options: 0.50 × 20, 0.33 × 30, 0.25 × 40, and 0.20 × 50. These combinations provided an ideal measure of an individual’s risk preference^11^.

**Figure 1:**
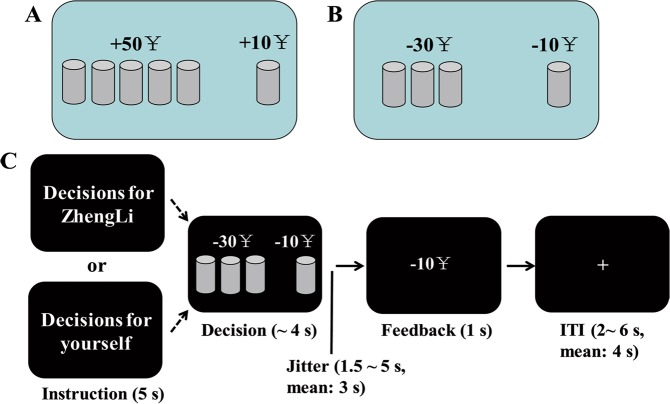
Experimental task and procedure. (**A**) An example of gain domain. The risky option leading to a probability of 20% of a ¥50 gain or not gaining anything is displayed on the left side of the screen. The sure option is displayed on the right side of the screen, leading to a sure gain of ¥10. (**B**) An example of loss domain. The risky option leading to a probability of 33% of a ¥30 loss or losing nothing is displayed on the left side of the screen. The sure option is displayed on the right side of the screen, leading to a sure loss of ¥10. (**C**) Timeline of each trial. Note that the instruction phase is presented only during the first trial in each block.

For each trial, one side of a screen was identified as the sure side where one cup was shown and ¥10 would be certainly gained (gain trial, Fig. 1A) or lost (loss trial, Fig. 1B). The other side of the screen was identified as the risky side, which showed an array of five, four, three, or two cups. Selecting one cup would lead to a designated number of money gained or lost, whereas the other cups would lead to no gain or loss. For a risky choice, a random process where *p* equals 1 divided by the number of cups was used to determine whether one cup selected led to a nonzero outcome^23^. To make the task easy to perform, participants were not asked to choose a specific cup in the risky option but were only asked to make a choice between a risky and sure option^11^.

The formal task consisted of four blocks (2 blocks for the self and 2 blocks for the other person). These blocks were counterbalanced in order across participants. At the start of each block, an instruction indicating decision target was presented for 5 s (Fig. 1C). In each trial, a sure and risky option were simultaneously displayed on a screen for 4 s, asking participants to make a choice by pressing the number key “3” to select the option presented on the left side of the screen, or by pressing the number key “4” to select the option presented on the right side of the screen. In every trial, the sure and risky options were randomly shown on the left or right side of the screen. The participants were asked to respond carefully but as quickly as possible (i.e., within 4 s), or otherwise they would get the worst possible outcome for that trial^11^. After the response and a delay ranging from 1.5 to 5 s (mean 3 s), feedback informing the participants of the outcome was presented for a period of 1 s. The intertrial interval (ITI) ranged from 2 to 6 s (mean 4 s). In each block, 45 trials were pseudo-randomly ordered, which comprised five times of repetition for each of the eight combinations and five null events (mean 2.5 s, ranging from 1 to 4 s). Each participant performed 180 trials in total.

A gender-neutral name (i.e., “ZhengLi”) was used to refer to the other person. This name was selected based upon a pilot study conducted on 20 additional participants (11 females, ages 20–27 years, *M* = 23.15, *SD* = 1.90). Participants were told that the other person was randomly selected from among the participants of another experiment. They were also informed that the other person would not make decisions for them. In actuality, as in previous studies^3,26^, the other person was a stranger and they would never meet.

Participants received a 50 RMB initial endowment in cash before the start of the experiment. They were told that, at the end of the experiment, the computer randomly selected one trial from the self trials, and any eventual gain or loss from the chosen trial would be added to or subtracted from the initial endowment. They were also informed that a payment would be made according to their actual choice. The participants would independently evaluate every choice because they were unaware of which trial would be selected^27,28^. Additionally, each participant also received a fee of 100 RMB for their participation.

The participants completed 18 practice trials before entering the scanner to acquaint themselves with the experimental procedure and task. After leaving the scanner, they were requested to answer a question: “Do you have any doubts that the decisions you made for the other during the task were real?”

### MRI data acquisition

Imaging data were acquired using a 3 T Siemens Trio scanner equipped with a 12-channel head coil. High-resolution T1-weighted anatomical images were acquired using a gradient-echo (MPRAGE) sequence (TR = 2530 ms, TE = 2.34 ms, flip angle = 7°, 192 sagittal slices, slice thickness = 1 mm, 1.0 × 1.0 × 1.0 mm voxel, FOV = 256 mm, matrix size = 256 × 256). T2*-weighted functional images were acquired using a gradient-echo echo-planar imaging (EPI) sequence (TR = 2200 ms, TE = 30 ms, flip angle = 90°, 36 axial slices, slice thickness = 4 mm, voxel size 3.4 × 3.4 × 4 mm, FOV = 220 mm, matrix size = 64 × 64).

### Data analysis

#### Behavioral data analysis

We conducted separate repeated-measures ANOVA on the proportion of times that the participants chose the risky option (i.e., risk rate) and reaction times (RTs), with decision target (self vs. other) and decision situation (gain vs. loss) as within-subject factors.

#### fMRI data analysis

The fMRI data were preprocessed and analyzed using Statistical Parametric Mapping 8 (SPM8, Wellcome Department of Cognitive Neurology, Institute of Neurology, London, UK). The first five volumes were discarded prior to analysis to allow for magnetic stabilization. Functional data were slice-time corrected to the middle slice. The functional images were then spatially realigned to the first volume to correct for head movement. Subsequently, the anatomical image was coregistered to the mean EPI image. The coregistered anatomical image was then segmented into gray matter, white matter, and cerebrospinal fluid using a unified segmentation algorithm^29^. The realigned functional images were normalized to the Montreal Neurological Institute (MNI) EPI template and resampled to 3 × 3 × 3 mm^3^. Finally, the normalized images were spatially smoothed using a Gaussian kernel with an 8 mm full width at half maximum.

At the first level of analysis, we modeled eight regressors of interest and convolved these with the canonical hemodynamic response function (HRF) on the basis of the general linear model (GLM). The eight regressors were defined according to target (self vs. other), situation (gain vs. loss), and choice (sure vs. risky). The defined regressors are as follows: (1) Participants made sure choices for the self in gain situations; (2) Participants made risky choices for the self in gain situations; (3) Participants made sure choices for the self in loss situations; (4) Participants made risky choices for the self in loss situations; (5) Participants made sure choices for others in gain situations; (6) Participants made risky choices for others in gain situations; (7) Participants made sure choices for others in loss situations; (8) Participants made risky choices for others in loss situations. The onset times were set at the start of the decision phase (i.e., when the two choice options appeared on the screen), and durations were calculated by subtracting the onset times of the decision phase from the feedback phase onset times. We added a parametric modulator to our GLM that scaled with reaction time (RT) with first order (linear) polynomial expansion. In addition, six motion-correction parameters were included as regressors of no interest to account for motion-related artifacts. High-pass temporal filtering with a cut-off of 128 s was applied to remove low-frequency drifts in signal. The GLM also considered signal temporal autocorrelations with a first-order autoregressive model to improve noise estimation^30^.

At the second level of analysis, the eight first-level contrast images from each participant were then analyzed in a full factorial design with target (self vs. other), situation (gain vs. loss), and choice (sure vs. risky) as separate factors. All results were reported using whole-brain familywise error (FWE) corrected (*p* < 0.05) at the peak level.

## Results

### Behavioral results

**Figure 2:**
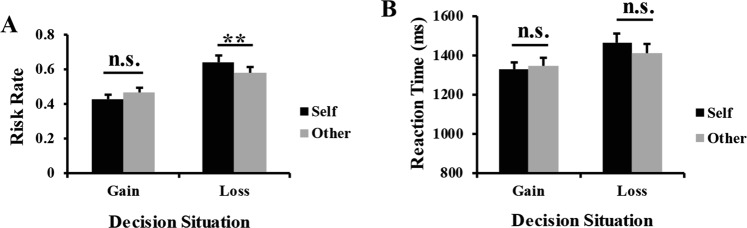
Significant interaction of target × situation for the risk rate (**A**) and reaction times (**B**). For the reaction times (**B**), the interaction was driven by greater differences between loss and gain situations in the decision-for-self condition than in the decision-for-other condition. Error bars indicate standard error mean. n.s. indicates nonsignificant differences as *p* > 0.05, **p < 0.01.

For the risk rate, the main effect of decision target was not significant, *F*(1, 24) = 0.22, *p* = 0.645, *η*_*p*_^2^ = 0.01. However, a significant main effect of decision situation was observed, *F*(1, 24) = 14.56, *p* = 0.001, *η*_*p*_^2^ = 0.38. The risk rate in loss situations (*M* = 0.61, *SD* = 0.17) was significantly higher than in gain situations (*M* = 0.44, *SD* = 0.12). Moreover, a significant interaction between decision target and situation was also observed, *F*(1, 24) = 9.75, *p* = 0.005, *η*_*p*_^2^ = 0.29, (Fig. 2A). Follow-up simple effect analysis revealed that the risk rate difference between decisions for the self (*M* = 0.42, *SD* = 0.15) and those for others (*M* = 0.47, *SD* = 0.14) was not significant in gain situations, *t*(24) = −1.19, *p* = 0.246. However, the risk rate was significantly higher when making decisions for the self (*M* = 0.64, *SD* = 0.18) than for others (*M* = 0.58, *SD* = 0.17) in loss situations, *t*(24) = 2.90, *p* = 0.008.

For RTs, the main effect of decision target was not significant, *F*(1, 24) = 0.24, *p* = 0.628, *η*_*p*_^2^ = 0.01. A significant main effect of decision situation was observed, *F*(1, 24) = 23.98, *p* < 0.001, *η*_*p*_^2^ = 0.50. RTs in loss situations (*M* = 1436.50, *SD* = 189.60 ms) were significantly longer than in gain situations (*M* = 1334.85, *SD* = 169.04 ms). Moreover, the interaction between decision target and situation was also significant, *F*(1, 24) = 7.50, *p* = 0.011, *η*_*p*_^2^ = 0.24, (Fig. 2B). Simple effect analysis showed that the RT difference between decisions for the self (*M* = 1327.48, *SD* = 167.52 ms) and decisions for others (*M* = 1342.22, *SD* = 214.42 ms) was not significant in gain situations, *t*(24) = −0.40, *p* = 0.692. Similarly, the RT difference between decisions for the self (*M* = 1462.64, *SD* = 221.02 ms) and decisions for others (*M* = 1410.36, *SD* = 215.32 ms) was also not significant in loss situations, *t*(24) = 1.21, *p* = 0.238. The interaction was driven by greater differences between loss and gain situations in the decision-for-self condition than in the decision-for-other condition, *t*(24) = 2.74, *p* = 0.011.

### fMRI results

#### Whole-brain analysis on the main effect

We found a stronger activation in the right anterior cingulate cortex (ACC), left dmPFC, and left insula (see Supplementary Table 1 for a complete list) when we compared decisions for the self to decisions for others (t-contrast: self–other). No significant activation for the opposite t-contrast (other–self) was found in the whole-brain analysis with the defined criteria. We found stronger activation in the left superior frontal gyrus when we compared decision making in gain situations with that in loss situations (t-contrast: gain–loss). The reverse t-contrast (loss–gain) revealed stronger activations in the right insula and left middle temporal gyrus. We observed higher hemodynamic activity in the left inferior temporal gyrus, left middle orbital gyrus, and left middle frontal gyrus (see Supplementary Table 2 for a complete list) when we compared the sure choices with risky choices (t-contrast: sure–risky). The reverse t-contrast (risky–sure) revealed stronger activations in the left dmPFC, bilateral AI, right caudate, and bilateral precentral gyrus (see Table 1 for a complete list).

**Table 1:** Main effect of choice (t-contrast: risky–sure).

Anatomical region		MNI coordinates			Voxels	*T*-value	*P*-value
		x	y	z			
AI	R	30	21	−8	172	9.11	0.000
Precentral gyrus	R	45	0	45	303	8.49	0.000
Caudate	R	9	9	3	302	8.48	0.000
AI	L	−27	21	−3	188	8.28	0.000
Middle occipital gyrus	R	27	−90	3	244	7.93	0.000
Middle occipital gyrus	L	−27	−90	−3	375	7.15	0.000
Precentral gyrus	L	−48	0	39	206	7.04	0.000
DMPFC	L	3	45	33	132	6.32	0.000
*ACC*	*L*	*−3*	39	27		*5.98*	*0.000*
Inferior parietal lobule	L	−27	−51	48	45	5.98	0.000

*Note*. AI = anterior insula, DMPFC = dorsomedial prefrontal cortex, ACC = anterior cingulate cortex, L = left hemisphere, R = right hemisphere. All results were reported using whole-brain FWE corrected (*P* < 0.05) at peak level.

#### Whole-brain analysis on target × situation interaction

**Figure 3:**
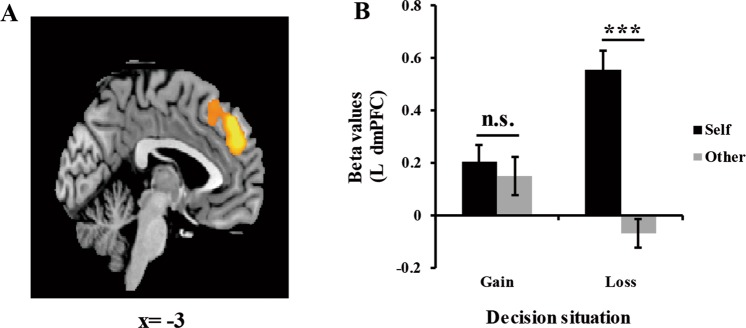
Interaction of target × situation in the left dmPFC. (**A**) BOLD response in the left dmPFC. (**B**) Beta values of the left dmPFC as a function of decision target and situation. Error bars indicate standard error mean. n.s. indicates nonsignificant differences as *p* > 0.05, ***p < 0.001.

**Figure 4:**
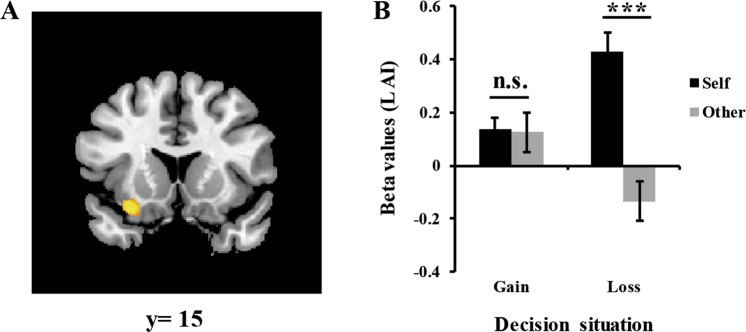
Interaction of target × situation in the left AI. (**A**) BOLD response in the left AI. (**B**) Beta values of the left AI as a function of decision target and situation. Error bars indicate standard error mean. n.s. indicates nonsignificant differences as *p* > 0.05, ***p < 0.001.

The *F*-contrast of target × situation interaction across choice revealed significant activations in the left dmPFC, bilateral AI, bilateral middle temporal gyrus (MTG), right ventromedial prefrontal cortex (VMPFC), right middle frontal gyrus (MFG), and bilateral thalamus (see Table 2). Beta values across left dmPFC and bilateral AI were extracted, and the data from sure and risky choices were merged by computing their average. Paired-sample *t* test revealed similar pattern of activations in the left dmPFC and bilateral AI. The activations in these three regions did not significantly differ between decisions for the self and for others in gain situations [left dmPFC: *t*(24) = 0.78, *p* = 0.445, Fig. 3A,B; left AI: *t*(24) = 0.13, *p* = 0.899, Fig. 4A,B; right AI: *t*(24) = 0.83, *p* = 0.416]. However, the significantly stronger activations in these three areas were observed when making decisions for the self than for others in loss situations [left dmPFC: *t*(24) = 11.20, *p* < 0.001; left AI: *t*(24) = 5.96, *p* < 0.001; right AI: *t*(24) = 6.50, *p* < 0.001].

**Table 2:** Significant interaction of decision target × situation.

Anatomical region		MNI coordinates			Voxels	*F*-value	*P*-value
		x	y	z			
DMPFC	L	−3	45	39	981	51.78	0.000
AI	L	−30	15	−15	60	46.26	0.000
MTG	R	66	−27	−9	104	44.21	0.000
VMPFC	R	12	60	3	71	41.30	0.000
AI	R	30	18	−12	42	35.58	0.000
MFG	R	48	21	42	25	30.38	0.003
MTG	L	−63	−33	−9	7	29.51	0.005
Thalamus	R	15	−18	6	3	28.71	0.007
Thalamus	L	−9	−12	3	9	27.01	0.013

*Note*. DMPFC = dorsomedial prefrontal cortex, AI = anterior insula, MTG = middle temporal gyrus, VMPFC = ventromedial prefrontal cortex, MFG = middle frontal gyrus, L = left hemisphere, R = right hemisphere. All results were reported using whole-brain FWE corrected (*P* < 0.05) at peak level.

#### Whole-brain analysis on target × situation × choice interaction

**Figure 5:**
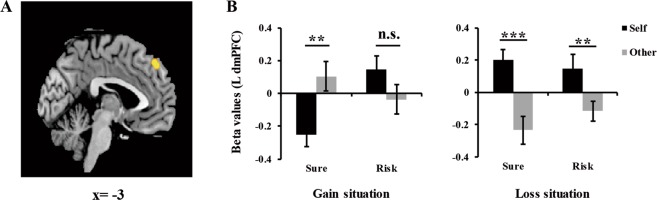
Interaction of target × situation × choice in the left dmPFC. (**A**) BOLD response in the left dmPFC. (**B**) Beta values of the left dmPFC as a function of decision target, situation, and choice. Error bars indicate standard error mean. n.s. indicates nonsignificant differences as *p* > 0.05, **p < 0.01, ***p < 0.001.

The *F*-contrast of target × situation × choice interaction revealed significant activations in left dmPFC (MNI: −3, 45, 45), right inferior temporal gyrus (MNI: 54, 9, −39), and right superior frontal gyrus (MNI: 18, 57, 36). We extracted the beta values of the left dmPFC. Paired-sample *t* test revealed that the activation in left dmPFC was stronger when the participants made sure choices for others than the self in gain situations [*t*(24) = 4.02, *p* = 0.001, Fig. 5A,B]; however, the brain activity in the left dmPFC did not show significant difference when the participants made risky choices for others than the self in gain situations [*t*(24) = −1.99, *p* = 0.058]. In loss situations, the activation in this region was both stronger when participants made sure and risky choices for the self than for others [sure: *t*(24) = 6.15, *p* < 0.001; risky: *t*(24) = 3.94, *p* = 0.001].

### Correlations

**Figure 6:**
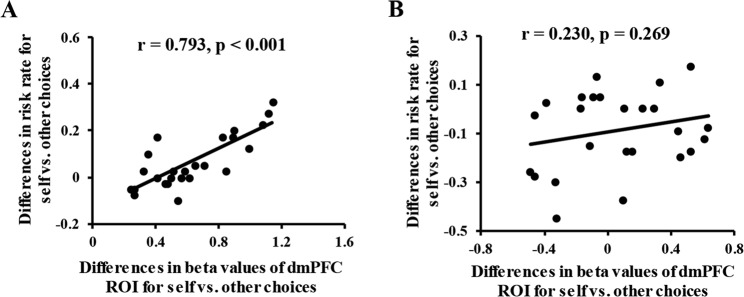
Scatter plots of correlation between the differences in risk rate and the differences in beta values of the dmPFC ROI for self versus other choices in gain and loss situations. (**A**) Differences in risk rate have a significant positive correlation with the differences in beta values of the dmPFC ROI for self versus other choices in the loss situation. (**B**) Correlation between the differences in risk rate and those in beta values of the dmPFC ROI for self versus other choices was not significant in the gain situation.

Correlation analysis on the dmPFC ROI determined by the whole-brain analysis on target  × situation interaction (i.e., that shown in Fig. 3) was implemented. Beta values of the dmPFC ROI was extracted, and the data from sure and risky choices were merged by computing their average. Subsequently, the differences in beta values of the dmPFC ROI in both gain and loss situations were computed by subtracting the “other” beta values of the dmPFC ROI from the “self”, and the differences in risk rate in both gain and loss situations were also computed by subtracting the “other” risk rate from the “self” risk rate. Finally, we examined the correlation between the differences in risk rate and the differences in beta values of the dmPFC ROI for self versus other choices in both gain and loss situations. The correlation analysis revealed that the differences in risk rate for self versus other choices have a significant positive correlation with the differences in beta values of the dmPFC ROI for self versus other choices in the loss situation (Fig. 6A), Pearson *r* = 0.793, *p* < 0.001. Thus, people with higher dmPFC activity for self versus other choices also display greater risky choices for self versus other in the loss situation. However, the correlation between the differences in risk rate and those in beta values of the dmPFC ROI for self versus other choices in the gain situation was not significant (Fig. 6B), Pearson *r* = 0.230, *p* = 0.269.

## Discussion

The present study investigated the influence of decision situation on neural responses to self–other decision-making under risk. Participants were more risk-seeking when making decisions for themselves than for others in loss situations but were equally risk-averse in gain situations. Consistent with this pattern, the stronger activations were observed in the dmPFC and AI when making decisions for the self than for others in loss situations but not in gain situations. Moreover, the activation in the dmPFC was stronger when people made sure choices for others than for themselves in gain situations but not when they made risky choices, and was both stronger when people made sure and risky choices for themselves than for others in loss situations. Our findings suggest that people are highly likely to differentiate the self from others when making decisions in loss situations, and thus shedding new light on self–other differences in decision making under risk.

A plausible explanation for our findings is that the dmPFC functions in evaluating the value of risky choice for self versus others. The activation in the dmPFC was both stronger when people made sure and risky choices for themselves than for others in loss situations, which indicates that people are more concerned with the outcomes of decisions for the self than for others in loss situations. This interpretation is supported by a previous study. Wang *et al*.^31^ found that the brain activity in dmPFC was both stronger for the self than others when participants played as actor (e.g., I hit someone.) and recipient (e.g., Someone hits me.) roles in negative interpersonal events. Our findings in loss situations stand in contrast to some previous studies that implicates dmPFC in calculating the value of choice for others^1,3,32^. For instance, Jung *et al*.^3^ showed that the activation in the dmPFC was stronger when making decisions for others than for self. A key difference between those previous studies and the present study is that the former used a gain task^1,32^ or a mixed gamble task^3^ that offered a variable chance of either gaining one amount of money or losing the same amount. In the present study, we adopted a task that can separate risky decision-making for gains and losses. Moreover, and importantly, risky decision making for gains and losses represents different psychological processes and may recruit separate neural structures^15,20,21^. Thus, such task differences could potentially account for the discrepancy in the overall findings between the studies. Our findings in gain situations support this conjecture by showing that the activation in the dmPFC was stronger when people made sure choices for others than for themselves in gain situations. Although decisions for others in gain situations would not immediately bring us economic rewards, they provide us with social rewards, such as another’s respect and admiration^33^.

The present study also revealed that the AI response was stronger when decisions were made for the self as compared with others in loss situations but not in gain situations. Our findings are in line with previous studies highlighting the key role of AI in signaling negative events, including aversive environments^34^, pain^35^, and financial loss^14,15,36^. Interestingly, our findings were also congruent with the evidence from human lesion studies indicating that patients with AI lesions show disrupted ability to use information about the probability of losses to update strategies in risky decision making^37^, as well as selective impairment in loss but not gain learning^36^. An alternative explanation for the present findings is that the stronger AI activation indicates greater loss aversion that is involved when making decisions for the self than for others in loss situations.

This explanation is supported by several other lines of evidence indicating that loss aversion is reduced when making decisions for others in a loss situation^38,39^. The risk-as-feelings hypothesis also suggests that people make risky decisions for themselves based on their subjective feelings toward risk, and that when they make risky decisions for others, they may base their decisions partly on their own feelings. Nevertheless, people may have difficulty fully empathizing with that person or considering the other person to have feelings that are as strong as their own^24,25^.

In sum, the AI may provide a fast and rough estimate for the potential of risky and sure options to result in an aversive outcome (i.e., a loss) on an emotional level. At the same time, this signal prepares the organism to take action to avoid the aversive outcome. Ishii *et al*.^40^ found that inactivating AI reduced risk preference in two types of gambling tasks, although AI inactivation did not affect decision making in risk-free control situations. They suggested that AI is causally involved in risky decision making and promotes risk taking. Thus, loss aversion is one mechanism believed to underlie an increase in risk-seeking behavior in loss situations.

Several potential limitations of this study merit comment. First, our study did not consider individual differences as a potential moderator. Individual differences in prosocial orientation^3,41^, impulsivity^42,43^, and anxiety^44^ may possibly affect the degree of self–other differences in decision making under risk. Future work is needed to examine the contribution of individual difference factors to our findings. Second, the present study did not exclude potential effects of learning from reward feedback^28^. It should be clarified in future research whether reward feedback has a different effect on risk preference for decisions for the self versus that of others.

Despite these limitations, our findings provide the first evidence that gain–loss situation modulates neural responses to self–other discrepancies in decision making under risk. We found that neural response in the dmPFC was stronger when people made sure choices for others than for themselves in gain situations, and was both stronger when people made sure and risky choices for themselves than for others in loss situations. The pattern of activations clearly indicates that our brain does not simply encode the difference between our own and another’s choice, but that it also depends on decision situation. In addition, AI response was stronger when decisions were made for the self than for others in loss situations but not in gain situations, which may indicate that the greater loss aversion was involved when making decisions for the self than for others in loss situations. Our work has implications for understanding addictive behavior and substance abuse. Our findings imply that the increased neural sensitivity to losses among individuals who are more risk-seeking and impulsive may result in maladaptive behavioral outcomes, such as substance abuse, drug seeking, or pathological gambling.

## Supplementary information

Supplementary Information
