# Supplementary material for: Gain–loss situation modulates neural responses to self–other decision making under risk
Source: Sci Rep. 2019 Jan 24;9:632. doi: 10.1038/s41598-018-37236-9 (PMC6345784; doi:10.1038/s41598-018-37236-9)
Supplement: Supplementary file 1 — Supplementary Information [file 41598_2018_37236_MOESM1_ESM.pdf]

Gain–loss situation modulates neural responses to self–other decision making under risk

Xiangyi Zhang, Shijia Li, Yongfang Liu, Xiyou Chen, Xuesong Shang, Fangzhu Qi, Xiaoyan Wang, Xiuyan Guo, & Jie Chen

Post-experiment self-report questionnaire

After the experimental task, participants were asked to answer a yes/no question:

“ Do you strongly doubt that the decisions for another person during the task were real? ”

A: Yes B: No

(used for the exclusion of participants; see *Methods, Participants*).

Supplementary Table 1. Main effect of decision target (t-contrast: self–other).

Anatomical region		MNI coordinates			Voxels	*T*-value	*P*-value
		x	y	z			
ACC	R	12	45	9	485	7.45	0.000
DMPFC	*L*	*-9*	*33*	*39*		*6.62*	*0.000*
Insula	L	-39	9	-12	21	6.06	0.000
Caudate	R	15	-15	21	5	5.42	0.002
MTG	R	69	-33	-15	33	5.30	0.004

Note. ACC= anterior cingulate cortex, DMPFC= dorsomedial prefrontal cortex, MTG= middle temporal gyrus, L = left hemisphere, R = right hemisphere. All results were reported using whole-brain FWE corrected ($P < 0.05$) at peak level.

Supplementary Table 2. Main effect of choice (t-contrast: sure–risky).

Anatomical region		MNI coordinates			Voxels	*T*-value	*P*-value
		x	y	z			
Inferior temporal gyrus	L	-54	-12	-27	253	7.79	0.000
Middle orbital gyrus	L	-3	48	-12	261	7.13	0.000
Middle frontal gyrus	L	-33	9	60	139	7.12	0.000
Superior temporal gyrus	L	-63	-27	9	211	6.67	0.000
Precuneus	L	-9	-57	21	136	6.11	0.000
Middle temporal gyrus	R	60	-3	-21	80	6.05	0.000

Note. L = left hemisphere, R = right hemisphere. All results were reported using whole-brain FWE corrected ($P < 0.05$) at peak level.
